# “I’d have no idea how to go about this…” - a survey of Australian medical students’ perspectives on their men’s health education

**DOI:** 10.1186/s12909-024-05045-6

**Published:** 2024-03-08

**Authors:** Zac E. Seidler, Ruben Benakovic, Michael J. Wilson, Jasmine M. Davis, Michelle Sheldrake, Margaret A. McGee

**Affiliations:** 1 Movember, Level 4/21-31 Goodwood St, 3121 Richmond, VIC Australia; 2 Orygen, Parkville, VIC Australia; 3 Centre for Youth Mental Health, The University of Melbourne, Victoria, Australia; 4 Australian Medical Students’ Association, Australian Capital Territory, Barton, Australia; 5 University of Melbourne, Victoria, Australia

**Keywords:** Gender, Medical education, Men’s health, Curriculum

## Abstract

**Background:**

While there have been calls over the last 15 years for the inclusion of training in sex and gender-based medicine in medical school curricula and to sustain such improvements through a more gender responsive health system, little progress has been made. A related objective of the Australian National Men’s Health Strategy (2020-30) is to improve practitioner core learning competencies in men’s health as a critical step to reducing the burden of disease in men and disparities between men in health care access and outcomes. The aim of this study was therefore to obtain Australian medical student perspectives on the extent to which men’s health and sex and gender-based medicine education is delivered in their curricula, their preparedness for engaging with men in clinical practice, and the men’s health content they would have found useful during their training.

**Methods:**

Eighty-three students (48% male) from 17 accredited medical schools, and in at least their fourth year of training, completed an online survey. The survey was co-designed by a multidisciplinary team of men’s health researchers and clinicians, alongside a student representative. A mix of quantitative and qualitative survey items inquired about students’ preparedness for men’s health clinical practice, and coverage of men’s health and sex- and gender-based medicine in their curricula.

**Results:**

Most students reported minimal to no men’s health coverage in their medical school education (65%). While few were offered optional men’s health units (10.5%), the majority would have liked more formal training on the topic (78%). Accompanying qualitative findings substantiated a lack of preparedness among medical students to engage male patients, likely stemming from minimal coverage of men’s health in their medical education.

**Conclusions:**

Australian medical students may feel underprepared for contemporary men’s health clinical practice, as well as, albeit to a lesser extent, women’s health clinical practice. There is a clear need and desire amongst medical students to enhance curricula with sex and gender-based medicine training.

**Supplementary Information:**

The online version contains supplementary material available at 10.1186/s12909-024-05045-6.

## Background

As men continue to die prematurely due to a number of largely preventable causes (e.g., heart disease, lung cancer, suicide) [1], timely and effective reach of health promotion efforts, and clinical care engagement, are critical to reduce the burden of disease in men [2–5]. While it is undoubtedly an ongoing health promotion challenge to ensure men access health services in a timely manner, it is growing increasingly clear that the health system, and the practitioners therein, experience challenges in reaching, responding to, and retaining men in care [6, 7].

A central consideration when designing solutions to these challenges is the role of gender, the complex interplay of social and cultural meanings and practices related to being a man or woman [8]. This is a distinct concept from sex which refers to biological and physiological attributes that distinguish males and females. These biological differences and social and cultural meanings and practices intersect with other sociocultural and environmental determinants in unique ways to affect health, healthcare experiences and health outcomes among men and women across different contexts [9–11]. These intersections, and their subsequent effects, have led to the formation of sex- and gender-based medicine framework, typically defined as the ways both biological and psychosocial context influence health and health outcomes [12].

For men, gender socialisation and traditional masculine norms such as emotional restriction, self-reliance, stoicism and risk-taking, have been shown to impede uptake and ongoing engagement in healthcare [6, 13, 14]. For instance, in mental health service contexts, uptake of psychotherapy has been labelled the ‘antithesis of masculinity’ [15]. Gender biases also influence how healthcare professionals view men and their health, with men frequently stereotyped as ‘bad patients’, delaying help seeking, refusing to openly communicate their concerns, and being less likely to engage in regular check-ups [16, 17]. In discussing health systems more broadly, Manandhar and colleagues [18] argued that “decision-makers in the global health system are not well-prepared to understand and effectively respond to, the structural, social, commercial and frequently gendered determinants of the major emerging burdens of disease” (p. 648). Qualitative evidence from Hale and colleagues [19] also suggests male general practitioners can be complicit in the co-construction of traditional masculinity as a hindrance to acceptable healthcare engagement. Specifically, male patients were labelled ‘inappropriate attender[s]’ (p. 707) if they were to transgress socialised self-reliance. Such biases may create dyadic challenges, meaning concurrent difficulties that arise for both patients and doctors within the healthcare setting. These dyadic challenges disrupt the patient-doctor interaction, potentially impacting men’s healthcare experiences and fuelling rigid attitudes and behaviours, misinformation, and mistrust in the therapeutic relationship [20–22]. A shift in the healthcare system’s approach to engaging men is therefore required to ensure those who overcome a plethora of help-seeking barriers, are met with a gender-sensitised, truly person-centred healthcare experience [23–25]. Seeking to explore and develop solutions to ensure health services can better attune to men and masculinities, in all their diversities, may serve to ameliorate the issue of sub-optimal health care interactions with men [26].

To improve the adaptability of contemporary health services and systems, practitioner workforces must be adequately attuned to the depth and diversity of men’s health experiences and use this knowledge to optimally engage with men around their health and wellbeing [5, 25, 27, 28]. Such a directive was outlined in the Australian Government’s second National Men’s Health Strategy (2020-30), with a central pillar focused on investment in the improvement of core learning competencies in men’s health knowledge, engagement, and male-centred practice of health professionals in primary care [29]. Without the actualisation of this update to health practitioner training, we risk a continuation of the status quo, whereby individual responsibility falls on the shoulders of male patients to adapt to a health system which often overlooks and/or misunderstands their needs [2, 30].

Health practitioner education and training that applies a sex and gender-based medicine framework recognises the unique but overlapping health system issues for men and women, allowing a similar approach to reform. There have been concerted efforts over the last 15 years, driven initially by the women’s health sector [9] and initiated by the National Institute of Health’s (NIH) Public Task Force on Women’s Health [5, 10] to formally embed sex and gender-based medicine education within curricula to ensure students have the necessary competencies in providing more equitable gender-sensitised health care for all people [27, 31, 32]. Echoing the value of co-design models of training development, such curricula reform must attend to student perspectives and perceived needs for future clinical practice. Holden and colleagues surveyed 170 medical students from one Australian University to assess the coverage of men’s health in their medical school education, and their resulting preparedness for providing care for men in practice [33]. Students reported men’s health content in their curriculum as being brief or absent, leading to a lack of preparedness for men’s health practice. However, this and other research examining men’s health coverage in curricula [34] and the broader men’s health training landscape, tends to focus on education and preparedness regarding men’s physical and reproductive health. Men’s health is more than just andrology, where there is now increasing recognition of the value of more holistic approaches to men’s health that include men’s social and emotional wellbeing and the impact and intersectionality of gender socialisation on men’s health [10, 35, 36]. The extent to which men’s health education has evolved to meet this definition, alongside the depth of coverage of sex and gender-based medicine in Australian Medical School curricula remain under-researched. There is therefore a need to understand medical students’ education experiences using this more contemporary lens of men’s health and sex and gender-based medicine to inform future curricula enhancement.

The aim of this study, therefore, was to undertake a survey of medical students attending Australian universities, to gain their perspectives on the extent to which men’s health and sex and gender-based medicine education is delivered in their medical school curricula, their perceived preparedness for engaging with men in clinical practice, and the particular men’s health content they would have found useful during their training.

## Methods

### Participants and procedure

A 24-item survey was co-designed by the multi-disciplinary authors who included a student representative of the Australian Medical Students’ Association (AMSA). The survey was made available online using the Qualtrics software and platform (Qualtrics, 2005; Provo, Utah, USA; Ver: XM; https://www.qualtrics.com) and promoted by the AMSA to its student representatives from each medical school, through medical student societies and medical schools directly. Eligible participants were students in at least their fourth year of medical training, attending one of the 21 Australian Medical Council (AMC) accredited medical schools or recent graduates and who provided their informed consent to undertake the survey. The sampling goal was to have students representing at least 75% of the AMC medical schools undertake the survey.

### Measures

Following completion of demographic information, medical students responded to quantitative and open-text qualitative questions across four topic blocks. For the first topic block, students were asked to respond regarding their understanding of men’s health, their understanding of sex and gender-based medicine, and the coverage of men’s health during their education. For this, students were asked to rate their understanding of men’s health and separately, their understanding of sex and gender-based medicine using a 4-point scale (‘*not at all*’ to ‘*thoroughly*’). They were also asked to provide their own definition of men’s health and, using a 4-point scale (‘*no coverage*’ to ‘*thorough coverage*’), asked to recall the coverage given to men’s health throughout their medical education.

The second topic block captured preparedness for men’s health practice. Using a 5-item measure previously employed by Holden et al. (2015) [33] and Henrich et al. (2012) [37], students were asked to rate their preparedness for working with men in clinical practice overall and in the provision of gender-sensitised care for men. Students rated each item on a scale from 1 (‘*not prepared*’) to 4 (‘*thoroughly prepared*’) and were able to provide further insight into their clinical preparedness for working with men through an optional open-text response.

The third topic block was on women’s health and contained 2 questions, namely, preparedness for women’s health clinical practice (using the same rating scale described above), and an optional open-ended question seeking further information on their preparedness.

The fourth block involved reflections on learning (5 questions). For this, students were asked to indicate whether dedicated electives were offered to them in men’s health, women’s health and gender and health throughout their degree (‘*yes*’, ‘*no*’, *‘cannot recall*’), whether they would have liked more education on men’s health, and more education on women’s health. For each of the latter two questions students were invited to provide details on what topics they would have found useful.

### Data analysis

Quantitative data were analysed using descriptive statistics in R version 4.2.1 - (R Core Team, 2022). Responses to the open-ended questions were analysed using inductive content analysis as described by Elo and Kyngäs [38]. This consists of three phases (preparation, organising, and reporting). Firstly, two researchers (MS, RB) engaged in preparation through reading all responses in detail, undertaking immersion in the data. Data were coded for manifest content whereby categories were developed to encompass similar responses and were purposely shaped to directly reflect participant responses rather than inferred underlying meaning or themes [39]. Codes and categories were generated to group like responses, and then subsequently collated into higher order headings through consultation. Finally, the higher order categories for each of the four open-text survey items was placed in a conceptual map to guide interpretation of the data (see Supplementary File 1).

## Results

Eighty-three students undertook the survey (75 students responded to all questions; 8 students partially completed the survey; Table 1). The desired sampling goal was achieved with respondents representing 17 of the 21 (76.2%) AMC accredited medical school programs. The demographic characteristics of survey respondents was comparable to data representative of Australian medical students more broadly, as reported for in 2022 from the Medical Schools Outcome Database (where the median age was reported as 25 years, the full cohort was 46.8% male, 86% domestic students, and 3.1% identified as a First Nations student) [40]. The qualitative and quantitative item results are presented together for each of the four survey topic areas.

[Cut]

**Table 1:** Student demographic profile

Profile characteristic		Survey participants (*n* = 83)
**Mean age** - **years** (SD, range; median)		23.9 (2.6, 21–36; 24)
**Gender** - *n* (%)		
	Man	40 (48.2)
	Woman	42 (50.6)
	Self-identified gender	1 (1.2)
**Country of birth** - *n* (%)		
	Australia	66 (79.5)
	Other country	17 (20.5)
**First nations identity** - *n* (%)		
	Yes	2 (2.4)
	No	81 (97.6)
**Year of medical degree** - *n* (%)		
	4th year	34 (41)
	5th year	28 (33.7)
	6th year	6 (7.2)
	Gap year/research year	2 (2.4)
	Recently graduated	13 (15.7)

### Understanding of men’s health and sex and gender based medicine

Survey results revealed that few students rated their understanding of men’s health as ‘*not at all*’ (*n* = 3; 3.6%) or ‘*thorough*’ (*n* = 3; 3.6%), with the majority reporting a ‘*somewhat*’ (*n =* 40; 48.2%) or ‘*moderate*’ (*n* = 37, 44.6%) understanding. For sex and gender-based medicine, the majority of students reported their understanding as ‘*minimal*’ (*n* = 32; 38.6%) or ‘*moderate*’ (*n =* 38; 45.8%), with only a few rating their understanding as ‘*not at all*’ (*n* = 5; 6%) or ‘*thorough*’ (*n* = 3; 3.6%).

Content analysis of open-text responses from 80 students revealed that 21 students (26.3%) defined men’s health through a fixed description of male-only health conditions, typically mentioning men’s sexual, urological and reproductive health (e.g., *“Health issues faced by men, usually relating to the male urinary and reproductive systems.”*). Fifty-five students (68.8%), offered a more holistic definition of men’s health, often referencing the intersecting roles of wellbeing, mental health, and/or sociocultural factors influencing health behaviours and health disparities. An emphasis on health care engagement and interaction was reinforced across some responses (e.g., “*the frequency and availability of health services to men*” and “*being cognisant of the fact that men often are avoidant of the healthcare system”*) rather than only specific disease groups. In addition, a female student’s definition emphasised interaction between health issues and the sociocultural context of men’s help-seeking: “*medical issues…which are especially important for men because they are poorly addressed/treated, have higher rates, or face stigma.”.* This was further complemented by definitions that touched on the role of *“various factors that may impact this [wellbeing] such as perception of the male gender in seeking help and shame of being vocal about mental health issues…”*.

### Coverage of men’s health education

No students reported thorough coverage of men’s health in their medical education. Twenty-nine students (34.9%) reported moderate coverage, 48 (57.8%) reported there being minimal coverage and six students (7.2%) reported no coverage at all. Only 8 of the 76 responding students (10.5%) recalled having the opportunity to take a men’s health elective or placement compared to 45 students (59.2%) who recalled the opportunity to take a women’s health elective or placement. Notable differences were found in the format in the instances where men’s health was covered (including formal case-based learning, to objective structured clinical examinations, and specific men’s health sensitive examination tutorials [e.g. prostate examination practice]).

Content analysis of open-text responses from all 80 students supported these findings, with students often reporting “*no formal teaching*” when asked to recall their education on men’s health. One male student recalled having “…*had two lectures on men’s health over the whole degree*,” while another claimed that men’s health only “…*briefly came up on my GP placement as my GP was very keen on it*.”

Andrology (male sexual and uro-reproductive health) was the most frequently reported men’s health education content, recalled by 50 students (62.5%), with majority of these reports relating to conditions of the prostate. While overall coverage was limited, some students nevertheless reported an over-representation of certain diseases and age groups of men, with one male student commenting on how they were taught “*common physical conditions in older men such as benign prostate hyperplasia. That’s all.*” Similarly, a female student noted how “*most of the men’s health discussions were around older men (50–60+) and did not necessarily focus on the specific needs of younger men.”*

More than a quarter of the students (*n* = 21, 26.3%) recalled teaching on men’s mental health and/or suicide risk. Only five students (6.3%) recalled some limited content on gender norms or masculinities and men’s health, including in relation to help seeking behaviours. A key trend appeared across these five responses, where students reflected being taught “*that men have a decreased likelihood of seeking help for medical concerns - decreased likelihood of opening up or seeking help for mental health”.* This gendered stereotype was perpetuated across university teachings for these students, with another female student recalling how “*in the rural program there was a single lecture involving a discussion of how men might be less likely to present with mood disturbances or health issues in general due to stoicism.”* Only three male students (3.8%) recalled brief content pertaining to considerations when engaging men in clinical practice with one student reporting on learning *“communicating and building rapport with male patients”, a* further mentioning *“gender roles when talking about history”*, and the third recalling content on *“…patients coming in with ticket of entry concerns concealing other health issues…so to be more investigative with men generally speaking”.*

### Preparedness for clinical practice

A majority of the medical students (*n* = 56, 63.9%) felt that they were ‘*moderately prepared*’ for working with men in clinical practice, with only a small number (*n* = 6, 7.2%) feeling ‘*thoroughly*’ prepared (Table 2). By comparison, 24 students (28.9%) felt thoroughly and 48 (57.8%) moderately prepared for working with women in clinical practice (Table 2.). While nearly two thirds of students reported being thoroughly or moderately prepared to reflect on their own gender assumptions and how they may influence their work with men, the majority of students reported being minimally or not at all prepared for (i) understanding how gender socialisation (“masculinities”) impacts groups of men differently, (ii) knowing what questions to ask to help a male patient explore the interaction between their experiences of masculinity and health or (iii) for applying strength-based care when engaging with men about their health (Table 2).

[Cut]

**Table 2:** Medical students self-rated preparedness

Item	Not prepared (*n*, %)	Minimally prepared (*n*, %)	Moderately prepared (*n*, %)	Thoroughly prepared (*n*, %)
Working with men	1 (1.2)	23 (27.7)	56 (63.9)	6 (7.2)
Working with women	0 (0)	7 (8.9)	48 (60.8)	24 (30.4)
Exploring interaction between men’s experiences of masculinity and their health	13 (15.9)	37 (45.1)	31 (37.8)	1 (1.2)
Applying strength-based care when engaging with men	13 (15.9)	44 (53.7)	21 (25.6)	4 (4.9)
Understanding the impact of gender socialisation on men	7 (8.5)	35 (42.7)	35 (42.7)	5 (6.1)
Reflecting on your own gender assumptions	6 (7.2)	23 (27.7)	44 (53.0)	9 (10.8)

Thirty-eight students provided further open-text insights regarding their clinical preparedness for men’s health. Students reiterated feeling unprepared for clinical practice, particularly in relation to applying a gender-based framework to optimally engage men, with students commenting that *“it’s often difficult to approach and we have had no formal teaching except for being told “men are difficult consumers*” and that “*the only specific training we have had is grossly generalised and often placed the problems with men’s health with the patient*”. A clear gap in training was outlined with some directly advocating *“there needs to be a part of the medical curriculum solely on men’s health - definitely lacking at this point.”* Masculinity and its intersections with health were frequently cited as gaps in students’ knowledge (e.g., *“While we usually do have males as patients in our cases, the relationship between masculinity and health is rarely, if at all, explored”*. Responses here linked this gap in knowledge to a lack of preparedness with students reflecting that they had “*Never been taught about masculinity and how to ask male patients about their experience of masculinity and their health or how this impacts them. I’d have no idea how to go about this…”*.

The difference in overall preparedness for men’s health compared with women’s health was stark, with this discrepancy further reinforced by a female student: *“As a woman I feel that while I may understand the clinical side of men’s health, I lack the insight to comfortably approach it with men and I feel that thus far my medical education has not filled this gap. I feel as though my male peers are much more prepared for women’s health than I am for men’s health.”*

For students that did report feeling prepared, they usually did not gain this preparedness through formal curriculum: *“A lot of these things weren’t inherently or explicitly taught in medical school, but I think I learned passively through experiences on clinical placement…”.* Even those students who did feel as though they had been taught appropriate information felt they “…*would struggle to apply this in a clinical context.”*

### Student interest in further men’s health education and training

The vast majority of students (*n* = 65; 85.5%) reported they would have liked more education and training on men’s health. In comparison, 40 students (53.3%) reported they would have liked more education and training on women’s health.

Regarding men’s health topics that students would have found useful, the three most common were men’s mental health (*n* = 33), gender, masculinity, and sociocultural aspects of men’s health (*n* = 25) and how to specifically engage and communicate with men (*n* = 22). Specific student suggestions included: *“…more specific teaching about approaches to male patients that accounts for barriers to care like reduced help-seeking and different forms of communication.”* and content on “*clinical soft skills - strategies to broach difficult topics such as mental health etc.*”, as well as *“mental health, gender identity, masculinity, lived experiences”*. Reflecting a common finding that young doctors are overburdened with mental health presentations, one male student was emphatic that he wanted training to work with men effectively and “*how to actually make a difference.”*

## Discussion

The results of this survey of medical students support and extend upon previous research emphasising the clear need for specific men’s health training in Australian medical school curricula in order to adequately prepare students for clinical practice with male patients [33, 41]. The current findings expand on this scant literature through detailed quantitative and qualitative examination of students’ experiences of men’s health coverage (that extend beyond an andrology focus) and their lack of preparedness for working with men in clinical practice. With more men seeking help due to effective health promotion campaigns, educational institutions now have the responsibility to ensure their graduates are confident and competent to effectively engage and respond to this increasing male clientele in their practice. Concerningly, this study found evidence for an inadequate level of content on men’s mental health, gender socialisation, and communicating and engagement with men in care, which left many students feeling considerably underprepared to work with men. This is despite long-standing recommendations for institutions to adopt a sex and gender-based approach to medical education [5, 9, 27]. In doing so, students would instead be provided with the core competencies necessary to deliver gender-responsive care and reduce the health inequities faced by men, women, and gender diverse people. Moving beyond the lack of content, this study also provided a platform for students from 17 different medical institutions to provide their voice on what men’s health topics would prepare them for clinical practice with men, which can be utilised to guide future medical curriculum development.

Overwhelmingly, students reported feeling underprepared for engaging with men in clinical practice, compared to their level of preparedness for engaging with women in clinical practice. This is consistent with the greater women’s health educational opportunities compared to that for men’s health reported by the students. Students reported a lack of preparedness on how to communicate with men using a strength-based approach and through an understanding of how gender socialisation impacts men, and how to explore the intersections of masculinity and health with their male patients. This lack of preparedness likely stemmed from the absence of training opportunities in men’s health throughout their medical degrees. Indeed, men’s health curriculum has historically focused on physical health, with a comparative scarcity of contemporary gender-based medicine approaches that consider broader sociocultural determinants of health and help-seeking. Students in this survey often defined men’s health inclusively in terms of sociocultural determinants of health and intersectionality between experiences of gender, culture, and sexuality. This suggests scope for medical curricula to significantly and urgently contemporise and adopt a comparable focus on men’s health beyond andrology alone.

Concerningly, some students reflected that their men’s health education highlighted homogenous, deficit-based narratives surrounding men’s engagement with their health and healthcare. If indeed students are being taught only about the challenges of engaging men, but not how to address and overcome these, what follows is an inherent perpetuation of these unhelpful tropes which can become entrenched over time in healthcare settings. This likely contributes to men’s poor uptake and retention in services and ultimately poorer clinical outcomes [2]. Conversely, students reflected that education regarding gender-sensitive practice strategies would have been useful. A gender and strengths-based approach to men’s health must be formally incorporated into the curriculum, emphasising the impact of sociocultural barriers and the intersections of masculinity and health, rather than perpetuating a rigid and non-constructive narrative, placing the locus of control purely on the individual man [42, 43].

Prior research has documented biases among health practitioners in viewing male help-seekers as reluctant or difficult patients [15, 22]. Results of this survey suggest gaps in medical education concerning the extent to which training is effectively targeting these biases. It is clear that a broader picture of ‘men as patients’ is required, as students in this survey recounted overly simplistic depictions of men as avoidant of services, or difficult to engage when they do present. In line with this, Griffith (2012) stressed that intersectional approaches to men’s health education are needed to create a more accurate reflection of the determinants of men’s health and to enable practitioners to respond effectively to boys and men in all their diversities [13]. It has long been argued that medical school curricula, in particular, should focus on gendered determinants of health communication, to optimise the effectiveness of practitioner-patient interactions and subsequent clinical outcomes [10, 11, 31, 32]. Encouraging emerging practitioners to expect more from their male clients than stereotypically reticent masculinities could be an important mechanism of change here.

Concordant with these findings, students commonly cited a need for content in their medical education focused on the communicative aspects of clinical practice, gender, and masculinity. This student experience compares with that reported by Rydberg et al. (2021) who provided evidence that medical students expect to receive training and develop competencies in sex and gender-based medicine [44]. Moreover, evaluation of higher education curricula must take account of the changing needs of students, emerging concepts and identify risks to the quality of the course of study. The findings of this survey provide viable avenues for needs-based curricula enhancement of tertiary medical curricula.

Alongside this, the quantity and content of men’s health teaching was varied, and much less frequent than women’s health teaching, which was reported often as having its own assigned block of lectures and tutorials as well as specific electives and placements. For students to have a deep understanding of sex and gender differences and how they impact health and health care, there needs to be improvements in both teaching and clinical exposure. Some student respondents noted much of their training in men’s health was incidental in nature, coming from chance encounters with supervising practitioners who held an interest in the area. Importantly, such passing down of informal learnings by practitioners, while well-intentioned, often lacks a structured, holistic, and strength-based men’s health underpinning and risks a snowballing effect across generations. Believing that men’s health, a complex topic impacting almost half of the population, is only worthy of ‘on the job’ vicarious teachings, underestimates the impact borne out in increasingly problematic men’s health outcomes, and ignores now longstanding advocacy including by consumers, for more attuned and gender-responsive services [e.g., 5, 26, 45, 46].

Previous research on the perspectives of academics and curriculum developers in Australia, albeit from one University only, found enthusiasm and desire for medical courses to increase the amount of men’s health content and gender-specific teaching [46]. The results presented here extend and reinforce this sentiment, evidencing the appetite for more comprehensive men’s health education among medical students from a broad range of institutions around Australia. However, almost a decade on, barriers such as an already overwhelmed curriculum, lack of guidance, oversight and relevant expertise, and lack of interest from clinical educators, have prevented many universities from implementing changes. These barriers no longer stand as impediments to curricula enhancement, having been superseded by a medical need for a gender responsive health system acknowledged by the global health sector [2, 47–49]. Moreover, internationally, robust curricula frameworks incorporating sex and gender-based medicine have been successfully devised [50, 51]. It is now incumbent on the men’s health sector to support and promote their implementation. Given the flexibility of modern pedagogic approaches that can exploit the increasingly available centralised digital repositories of expert developed teaching resources available to streamline enhancement, the opportunities for widespread reform are clear. The Royal Australian College of General Practitioners offers, within its curriculum, an optional contextual unit on men’s health that delivers dedicated content through a more holistic lens, including a focus on sociocultural and gendered (masculinity) determinants of access and uptake and approaches to communicating with men to optimise their engagement and health care outcomes. This corresponds to the type of content that medical students are seeking early in their education, therefore building this syllabus out through co-design, technological advancement and extending the dissemination to medical students during their foundation learning would enhance preparedness for practice with men.

In light of the current findings, several limitations must be considered. Whilst the sampling goal of obtaining representation from at least 75% of Australian Medical Schools was achieved, the relatively small sample size of 83 students likely limits the generalisability of the findings. The demographic profile of the participating students was however comparable to that for all medical students graduating from Australian universities in 2022 [40]. In addition, this survey may have attracted responses from those with strong views and interest in men’s health, creating a potential bias in our results towards those who wish to see more men’s health content within their curricula. In designing the study, it was determined that offering a payment for participation would help to minimise this potential bias. Despite these constraints, the content analysis of open-text responses saw a high frequency of overlap across a diverse sample of students, suggesting the questions tapped into a shared learning experience across institutions which may extend beyond this sample. This survey also relies largely on student recall of coverage of sex- and gender-based medicine in their curricula, which is likely limited by recall bias. The students’ recall of limited men’s health content does however align with a recent review of the course summaries and learning outcomes of a sample of 10 Australian medical school curricula, where no dedicated men’s health courses and no specific reference to men’s health were found (Seidler et al., PhD, unpublished data, December 2023). In addition, given the online survey format, responses were likely influenced by participants’ subjective interpretation of the meaning of ‘men’s health content’; as such, we may not have captured the full breadth of included content in current curricula. Where possible however, questions and prompts in the survey were included to guide students’ understanding of the curricula under study here. Notwithstanding this, in-depth qualitative interviews or focus groups would be a worthwhile methodology to apply in future, to capture student perceptions and understandings of men’s health curricula (or lack thereof) more fully. Finally, the authors acknowledge that gender is not a binary and the focus on men’s health education, and for comparison women’s health education, in this study, was due to the limited existing research on non-binary people and catering to their needs in health settings.

## Conclusions

Overall, this study highlights that many medical students define men’s health in a holistic sense, inclusive of mental health, wellbeing, and sociocultural influences. However, this is often at odds with their teaching, with many students feeling underprepared to engage with and respond to help-seeking men. Students may be forced to seek education outside of their official curriculum, reinforcing the likely value of inclusion of more comprehensive men’s health content in their medical training. The current curricula focus on andrology, and the over-reliance on stereotypes of men as ‘difficult patients’ to frame teaching, reinforces a need for the medical curriculum to be updated and streamlined to be more reflective of medical students’ holistic views of men’s health. Men’s health educators should now respond to the clear desire among students for more comprehensive gender-sensitive, competency-based training, that could serve to improve their confidence and competence to effectively reach, respond to and retain men in health services.

### Electronic supplementary material

Below is the link to the electronic supplementary material.

Supplementary Material 1

## Data Availability

The anonymised data that support the findings of this study may be available from the authors upon reasonable request. Please contact the corresponding author (Zac E. Seidler, zac.seidler@movember.com) for all data availability enquiries. As the approved protocol for this study did not include extended use of the data for further research, requesting users will need to have independent human research ethics approval for data access.

## Abbreviations

AMSA: Australian Medical Students’ Association
AMC: Australian Medical Council
