# Supplementary material for: “I’d have no idea how to go about this…” - a survey of Australian medical students’ perspectives on their men’s health education
Source: BMC Med Educ. 2024 Mar 8;24:260. doi: 10.1186/s12909-024-05045-6 (PMC10924408; doi:10.1186/s12909-024-05045-6)
Supplement: Supplementary file 1 — Supplementary Material 1 [file 12909_2024_5045_MOESM1_ESM.docx]

**Supplementary Table 1.** Conceptual Map of higher-order categories.

| **Open-text Qualitative Item** | **Higher-order categories** |
| --- | --- |
| How would you define MEN'S HEALTH? (*n=80*) | Holistic health (*n*=55, 68.8%) |
|  | Broad definition of male relevant health problems (*n*=21, 26.3%) |
| If you recall coverage of MEN'S HEALTH in your medical school education, please briefly describe the subject matter. (*n*=80) | Andrology (*n*=50, 62.5%) |
|  | Men’s mental health and/or suicide risk (*n*=21, 26.3%) |
|  | Gender norms or masculinities and men's health (*n*=5, 6.3%) |
|  | Engaging men in clinical practice (*n*=3, 3.8%) |
| How prepared do you feel, from your medical school education thus far, for working with men in clinical practice? (*n*=38) | little to no teaching, not well taught, very generalised (*n*=18, 47.4%) |
|  | Not prepared (*n*=13, 34.2%) |
|  | masculinity or masculinity & health not covered (*n*=13, 34.2%) |
|  | Prepared (*n*=10, 26.3%) |
| What MEN'S HEALTH topics would you have found useful? (*n*=62) | Men's mental health (*n*=33, 53.2%) |
|  | Gender, masculinity, and sociocultural aspects of men's health (*n*=25, 40.3%) |
|  | How to communicate and engage men (*n*=22, 35.5%) |
|  | Physical conditions in males (*n*=13, 21.0%) |
|  | Education on sex/gender differences (*n*=9, 14.5%) |
|  | Reproductive (*n*=6, 9.7%) |
|  | Bias and stigma (*n*=2, 3.2%) |
